# Bilateral Stroke: A Case Report

**DOI:** 10.7759/cureus.50770

**Published:** 2023-12-19

**Authors:** André Mendes, Emanuel Fernandes, Ana P Rezende, Jerina Nogueira, Augusto Mendonça

**Affiliations:** 1 Internal Medicine, Unidade Local de Saúde do Norte Alentejano - Hospital Dr. José Maria Grande, Portalegre, PRT

**Keywords:** ischemic stroke, atypical symptoms, altered mental state, brain ct scan, bilateral stroke

## Abstract

We present the case of a 74-year-old woman, functionally independent in her daily activities, with a medical history notable for hypertension and dyslipidemia. She presented to the emergency room with an altered level of consciousness, opening her eyes only to pain, no verbal response, and flexion withdrawal from pain coupled with a Glasgow Coma Scale of 7. The intensive care unit was promptly summoned, and the patient was intubated and admitted to intensive care. Comprehensive laboratory assessments revealed no abnormalities and an initial cerebral CT scan showed no acute changes. A subsequent CT scan performed 24 hours post-event disclosed bilateral ischemia affecting the territories of the anterior and middle cerebral arteries. Regrettably, this catastrophic event precluded any potential for recovery. Consequently, the decision was made not to pursue further investigations to determine the underlying cause. The medical team opted for supportive treatment and comfort measures. Tragically, the patient died on the 37th day of hospital admission.

## Introduction

Cerebrovascular diseases are prevalent, and individuals experiencing strokes frequently seek emergency care. Recognizing symptoms and diverse presentations is crucial for prompt diagnosis and appropriate treatment, given the advent of novel therapies. While conventional signs such as dysarthria, facial paralysis, and unilateral limb weakness are well-known, strokes may manifest solely through altered mental status, impeding accurate neurological assessments [1-3].

Several cases of stroke presenting with altered consciousness have been reported mainly affecting thalamic regions and other cases, when, at the same time, the patient has an infection. Unilateral strokes in a large area of the brain can also provoke altered mental status through brain edema [4].

In Portuguese hospitals, when stroke protocols are activated, the patient is evaluated to exclude other conditions, brain CT is done to differentiate hemorrhagic events from ischemic strokes and then, if the patient meets the criteria, thrombolytic treatment is started. In better-condition patients, a complementary study is also done to find and treat the cause of the stroke to prevent further events. In this case, as the patient deteriorated rapidly, only a basic study was done with EKG, blood analysis, and brain angio-CT.

Another problem that clinicians face, is that there are countless causes of altered mental status, and some, more frequent than stroke-like, infections, metabolic disorders, toxics, endocrine disorders, and psychiatric conditions.

## Case presentation

**Figure 1:**
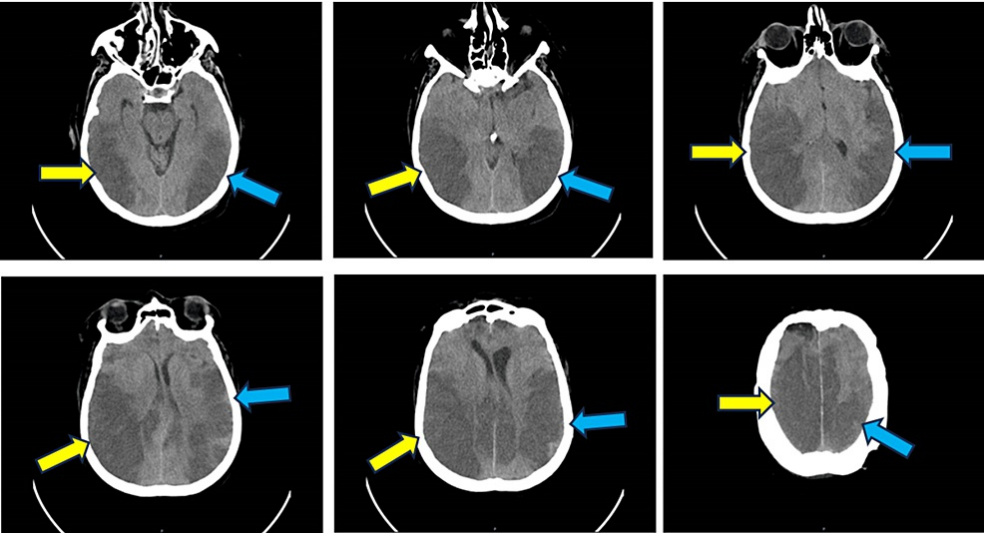
CT scan of a bilateral stroke with ascending cuts of the ischemic area in the right hemisphere (yellow arrow) and left hemisphere (blue arrow)

A 74-year-old autonomous woman, with a medical history of hypertension and dyslipidemia, was admitted to the emergency room due to an abrupt decline in mental status, presenting a Glasgow Coma Scale of 7 with two hours of evolution. Upon examination, the patient exhibited an inability to respond to queries or follow commands. Cardiac and pulmonary auscultation revealed no abnormalities, blood pressure was 137/89 mmHg, and cardiac frequency was 92 bpm. As the patient was comatose, neurological examination results must be taken carefully. At admission the calculated National Institutes of Health Stroke Scale score was about 20, with movements only to pain, aphasic, not performing tasks, without horizontal extraocular movements, impossible to evaluate visual fields, without facial palsy, not moving arms or legs, impossible to test language, sensation and extinction. The altered consciousness, coupled with a Glasgow Coma Scale of 7, which made it unable to maintain a clear airway, prompted intensive care intervention, including intubation. A subsequent brain CT scan disclosed no acute lesions. Brain and neck angio-CT did not reveal an embolic cause. Laboratory evaluation excluded other major causes of impaired consciousness, infections, electrolyte imbalances, hypo and hyperglycemia, liver and renal failure, drug overdose, alcohol intoxication, carbon monoxide intoxication, and thyroid disorders. EKG revealed a sinus rhythm. The patient was admitted to the ICU, receiving perfusion of fentanyl and propofol. A repeat brain CT scan at 24 hours revealed extensive bilateral ischemic hypodensity in the temporal-occipital, parietal, and posterior frontal cortico-subcortical areas with effacement of sulci and slight repercussion in the lateral ventricles without deviations from the midline, without associated hemorrhagic foci and conflict in the occipital foramen (Figure 1).

Despite efforts, the patient's neurological status deteriorated, leading to a grim prognosis. On day seven, the patient was diagnosed with bilateral ischemic stroke of the anterior and middle cerebral arteries, exhibited a Glasgow Coma Scale of 7, isochoric and isoreactive pupils, oculocephalic and corneal reflexes, and limb hypotonia, and was transferred to internal medicine care. The decision to forego further investigations due to the reserved prognosis was made, and the patient died on day 37 of admission.

## Discussion

Patients with neurological changes are not rare in the Emergency Department. In fact, in the year 2017, there were around one million new diagnoses of stroke in the European Union, with almost 10 million people living after the event [5]. These numbers have allowed hospitals and other organizations to invest in this medical area and now, most Portuguese hospitals have protocols regarding the management and treatment of strokes. The problem is that these protocols are only activated when the patient presents with dysarthria, facial paralysis, and muscle weakness which brings to question how many patients we are forgetting.

Coma is known to be a symptom of stroke and cerebrovascular diseases account for almost 50% of coma presenting in the Emergency Department, mostly in intracerebral hemorrhage but can be seen also in ischemic events, like the occlusion of the basilar artery. Coma in ischemic stroke is due to one of two causes, the most frequent is a massive stroke that provokes brain-stem compression caused by herniation and infarct swelling leading to deviation of brain midline affecting intact brain and the other is a rare bilateral stroke [6].

At this point, we know that patients presenting with a reduced level of consciousness are at a major risk of missed or delayed diagnosis, which will result in patients not receiving acute treatment as it is time-dependent with the worst outcomes [7].

This case underscores the challenge of diagnosing strokes when presenting with altered mental status. While conventional symptoms guide prompt treatment, atypical presentations may hinder timely intervention. This prompts the need for heightened awareness regarding strokes in patients exhibiting altered mental status.

## Conclusions

Numerous cases report strokes initially presenting as altered mental status. The atypical nature of these presentations often results in suboptimal treatment. Therefore, advocating for increased awareness of the potential association between altered mental status and strokes is imperative to optimize patient outcomes.
